# Enhanced Fat Graft Viability and Remodeling Using a Helium-based Radiofrequency Device to Prepare the Recipient Site

**DOI:** 10.1007/s00266-023-03749-6

**Published:** 2023-12-14

**Authors:** Paul G. Ruff, Aris Sterodimas

**Affiliations:** 1 West End Plastic Surgery, MedStar Georgetown University, Washington, DC USA; 2 Department of Plastic and Reconstructive Surgery, Metropolitan General Hospital, Athens, Greece

**Keywords:** Fat graft retention, Adipose-derived stem cells, Helium plasma, Radiofrequency, Bolus fat graft, Autologous fat graft

## Abstract

**Background:**

Improvements to autologous fat grafting for soft tissue augmentation are needed to overcome the unpredictable volume retention. Approaches such as fat harvesting and processing, injection technique, preparation of the recipient site, and supplemental biologics are topics of ongoing research. Here, an energy-based device was investigated as a stimulatory tool for recipient site preparation for improving fat graft retention.

**Objective:**

The objective was to measure the stimulatory responses in fat grafts after 4 weeks when using a helium-based radiofrequency device to pretreat the recipient tissue.

**Methods:**

Using an autologous fat grafting mouse model, the inguinal fat pad was grafted in a small cranial pocket after either a saline injection alone (control) or a saline injection followed by pretreatment (treated). The fat pad was resected after 4 weeks, sectioned and stained with immunofluorescence markers to investigate tissue remodeling.

**Results:**

Pretreatment resulted in higher viability of adipocytes, a higher concentration of viable ASCs in areas of adipose tissue regeneration, and localized macrophages in the areas of regeneration when compared to the control. There was no observable difference in vascularity or angiogenesis. The staining for ASCs was higher in the pretreated group in comparison with the control group (5.0% vs. 3.3%, p=0.36) when using a pixel classifier in QuPath in the viable adipose tissue regions.

**Conclusions:**

The use of a helium-based radiofrequency device as a pretreatment tool appears to increase the viability of the adipose tissue likely due to higher concentration of ASCs. The apparent increase in viable ASCs may be due to enhanced proliferation or paracrine recruitment of these cells in response to the helium-based radiofrequency treatment.

**No Level Assigned:**

This journal requires that authors assign a level of evidence to each submission to which Evidence-Based Medicine rankings are applicable. This excludes Review Articles, Book Reviews, and manuscripts that concern Basic Science, Animal Studies, Cadaver Studies, and Experimental Studies. For a full description of these Evidence-Based Medicine ratings, please refer to the Table of Contents or the online Instructions to Authors www.springer.com/00266.

Bullet List of Important Points:

- Pretreatment of the fat graft recipient site increases the viability of the adipose tissue after 4 weeks in comparison with the control grafts.
- The increased viability is likely due to the observed increase in adipose-derived stem cells in the pretreated group.
- Pretreatment enhanced the adipose tissue remodeling as colocalization of adipose-derived stem cells and macrophages showed an active remodeling, whereas the control group exhibited more necrotic and fibrotic tissue.

**Supplementary Information:**

The online version contains supplementary material available at 10.1007/s00266-023-03749-6.

## Introduction

Widespread adoption of autologous fat grafting has led to numerous reconstructive, esthetic, and regenerative applications [1–3]. The benefits of autologous fat grafting include its inherent biocompatibility, low cost, accessibility, and low immunogenicity. Due to its myriad uses, surgeons from different medical specialties developed new techniques geared toward increasing fat retention. These developments in fat harvesting, fat processing, preparation for the donor site and the recipient site, and fat grafting techniques have improved patient outcomes. For soft tissue augmentation, these techniques enhance fat retention by increasing fat graft survival. However, there is no standardized method for autologous fat grafting leading to unpredictability and variable rates of fat retention.

Strategies to enhance autologous fat graft retention aim to increase angiogenesis into the graft and the proliferation of progenitor cells [4]. Grafted adipose tissue loses volume as mature adipocytes undergo apoptosis within the first 72 hours due to a lack of oxygen and nutrients [5]. Adipose-derived stem cells (ASCs) are more resistant to these hypoxic conditions and begin replacing the dead adipocytes after these older cells are removed by macrophages. The new blood vessels provide the cells, nutrients, and growth factors to support this remodeling of the fat graft. Adipose tissue that remains without oxygen for too long becomes necrotic leading to tissue fibrosis and cyst formation. Approaches to overcome poor retention for soft tissue augmentation increase tissue oxygen levels, the survival and number of progenitor cells, and growth factors in the recipient site. The main strategies include physical manipulation of the recipient site (e.g., BRAVA®, microneedling, biostimulation of tissue) [6, 7], or injection of either botulinum toxin for facial fat graft [8], growth factors [9–11], or progenitor cells during the fat graft, i.e., cell-assisted lipotransfer (CAL) [12]. Supplementation of autologous fat grafts with ASCs [13], platelet-rich plasma (PRP) [14], stromal vascular fraction (SVF) [15], or exosomes [16] either alone or in combination have increased fat graft retention [17]. These techniques lead to increased vascularity within the graft and increased proliferation and number of ASCs.

An uncommon technique for soft tissue augmentation is the use of energy-based devices for recipient site preparation prior to grafting the fat. While ultrasound and laser devices are successfully used for removal of fat [18, 19], energy-based devices have rarely been investigated for their tissue stimulating properties regarding recipient site preparation. Tissue biostimulation using a subdermal fractional CO_2_ laser was utilized by Jianu et al. to improve patient outcomes from facial fat grafting. The approach uses low-level laser therapy, hypothesized to induce ASCs to release proangiogenic and proliferative factors as observed in vitro [7]. The resulting photobioactivation changes the redox state of the ASCs and likely the surrounding tissue [20].

Renuvion (Apyx Medical Corporation, Clearwater, Florida), a helium-based radiofrequency device that combines radiofrequency (rf) energy and helium plasma, may be another approach to stimulate the tissue by combining their two effects: promotion of angiogenesis through heating the tissue, and the proliferation of ASCs and paracrine recruitment of immune cells in response to plasma treatment [21–25]. Furthermore, plasma treatment in mouse wound models was shown to augment tissue oxygenation [26]. The combination of these effects may prove to be another strategy to promote fat graft retention.

For this study, we hypothesized that pretreatment of the recipient site with a helium-based radiofrequency device stimulates the tissue to enhance fat graft retention. To investigate this, the viability, vascularity, and the presence of macrophages and ASCs in the fat graft were stained in FFPE slides at 4 weeks in pretreated mice in comparison with the controls. Given the anecdotal evidence for plasma-enhanced recruitment of immune cells, enhanced proliferation of ASCs, increased tissue oxygenation, and promotion of angiogenesis, it was hypothesized that pretreatment of the recipient tissue would enhance fat grafting of bolus fat grafts. We observed more viable adipose tissue in the pretreated group in comparison with the control mice. Additionally, the overlapping location of ASCs with macrophages in the pretreated group suggested increased remodeling activity of the dead adipose tissue. The pretreatment did not show any evidence for enhanced angiogenesis or increased secretion of vascular endothelial growth factor (VEGF). Furthermore, increased fibrosis and damaged adipose tissue morphology were observed in the control group.

## Materials and Methods

The study was conducted at the Comparative Medicine facility at the University of South Florida (USF) and approved by the USF’s Institutional Animal Care and Use Committee (IACUC). The paraffin embedding, sectioning and immunofluorescence staining were performed by the Molecular Pathology Core at the University of Florida. Whole slide imaging performed by the Analytic Microscopy Core Facility at H. Lee Moffitt Cancer Center.

### Mouse Model for Autologous Fat Grafting

The animals used in this study were cared for in accordance with the USF institutional guidelines. Fifteen mice (Eight-week-old female C57BL/6, Jackson Labs) were split into the following three study arms: control, pretreated, and baseline. The control and pretreated mice were anesthetized with 2% isoflurane, whereas the baseline mice were euthanized under CO_2_ for initial fat pad collections. Following collection, the inguinal fat pads were weighed, and the donor sites were sutured closed in both the control and pretreated groups. The fat pad (85 mg on average) was then inserted into the recipient site which followed the cranial fat graft model as published by Kato et al. [27]. Briefly, a small pocket was made on top of the cranium through a small incision (5 mm long) and expanded using forceps. The inguinal fat pad from the same animal was then inserted into the recipient site after either injection of 0.1 ml of saline (control), or after injection of 0.1 ml of saline followed by pretreatment (helium-based rf device). Mice were euthanized 28 days later, and the grafted fat pad was excised and weighed. The baseline, control, and pretreated group fat pads were fixed in 10% neutral buffered formalin (Fisher Scientific) for 24 hours, then washed twice with 1X PBS and embedded in paraffin for immunofluorescence staining and assessment.

### Image Acquisition

Fluorescence-labeled slides were scanned at 20X (0.5 mm) with an Akoya Fusion slide scanner equipped with fluorescence LED light source and DAPI, OPAL520, OPAL570, and OPAL690 filters. The same acquisition settings were used among slides within each staining panel. Whole slide images were created in QPTIFF format.

### Immunofluorescence Assessment

The fat grafts in this study were resected and compared at 4 weeks post-graft to assess energy-based pretreatment as a recipient tissue preparation technique. Viable adipocytes and ASCs, macrophages, and the vascularity were visualized by immunofluorescence staining for perilipin and CD44, MAC2, and CD31, respectively. After preparing 4-mm-thick sections from the paraffin-embedded sections, the slides were stained with the following primary antibodies at 4°C overnight: guinea pig anti-mouse perilipin (Progen, 1:100 dilution), rat anti-mouse CD44 (Invitrogen, 1:200 dilution), goat anti-mouse CD31 (R&D Systems, 1:25 dilution), rabbit anti-mouse VEGF (Invitrogen 1:100 dilution), and rat anti-mouse MAC2/Galectin 3 (Invitrogen, 1:100 dilution). The following secondary antibodies were used at room temperature for triple fluorescence staining: Alexa Fluor 488-conjugated donkey anti-guinea pig immunoglobulin G (Jackson ImmunoResearch Labs, 1:500 dilution), Alexa Fluor 647-conjugated donkey anti-rat immunoglobulin G (Jackson ImmunoResearch Labs, 1:500 dilution), Cyanine3-conjugated donkey anti-goat immunoglobulin G (Jackson ImmunoResearch Labs, 1:500 dilution), Cyanine3-conjugated donkey anti-rabbit immunoglobulin G (Jackson ImmunoResearch Labs, 1:500 dilution). A slide with the three associated secondary antibody staining was used as a negative control for each triple fluorescence-stained slide. Nuclei were stained with DAPI (Invitrogen, 1:500 dilution). All images were spectrally unmixed in Phenochart. The ASC quantification was done in QuPath using a pixel threshold classifier after annotating the viable adipocyte region in each mouse tissue.

## Results

### Helium-based Radiofrequency Pretreatment Increased Adipose Viability

**Fig. 1.**
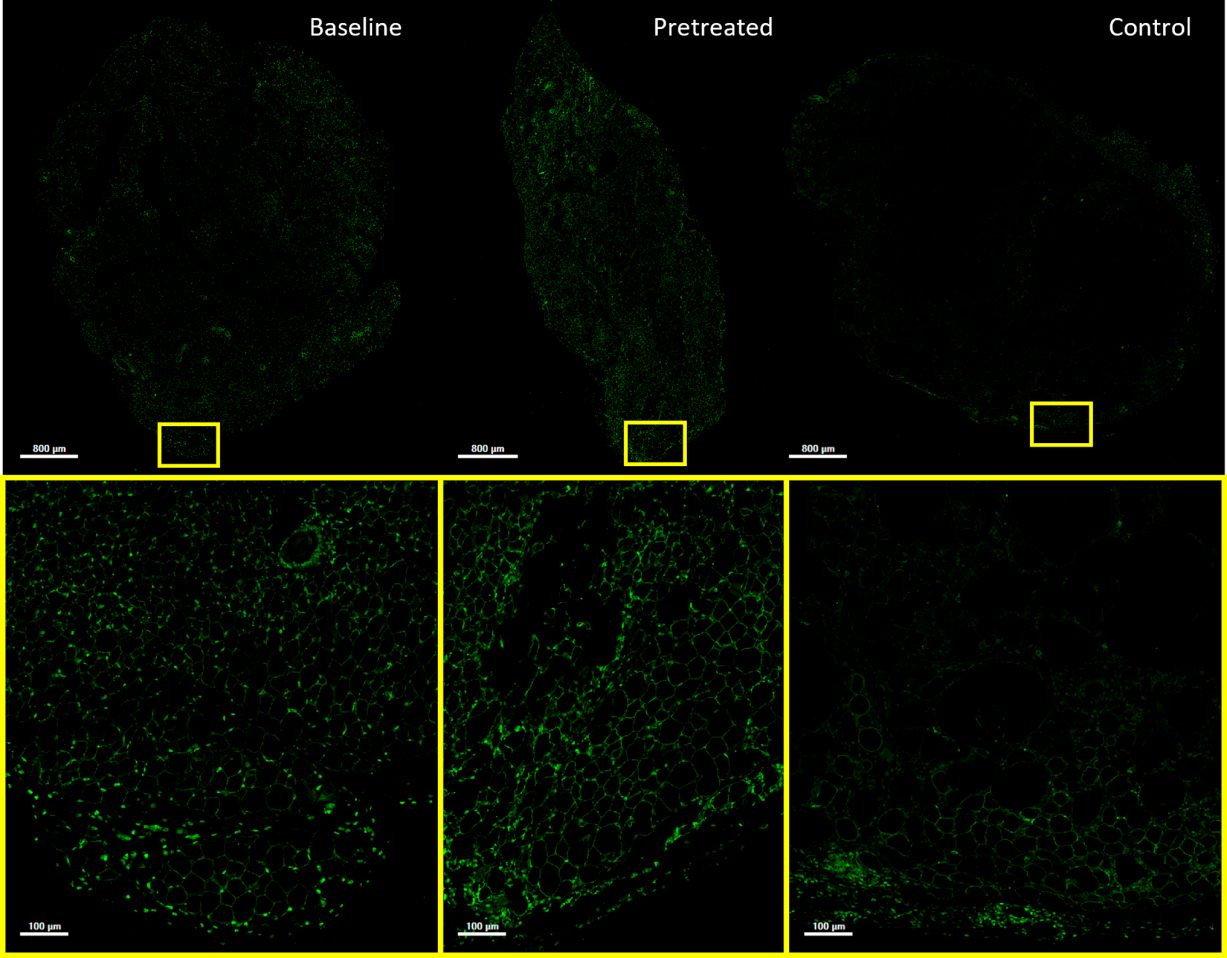
Helium-based radiofrequency pretreatment enhances the viability of the adipose tissue in comparison to the control. The sectioned tissue stained with perilipin to assess viability is shown for the baseline (day 0, left), pretreatment (day 28, middle), and control (day 28, right). The top row are whole slide images (scale bar = 800 mm), and the bottom row are 10x sections (scale bar = 100 mm) denoted by the yellow box. Pretreatment enhances the viability at 28 days in comparison to the control. The morphology and presence of ASCs in the pretreated grafts are more similar to the baseline than the control.

At baseline, the adipose tissue is healthy with uniformly sized and distributed adipocytes as shown in Fig. 1. The perilipin positive stain in the cytoplasm denotes viable adipose tissue, and the smaller, strongly perilipin positive cells are the preadipocytes, or ASCs. After four weeks post-procedure, the control and pretreated grafts are in the middle of the remodeling phase; the older non-viable adipocyte tissue is recycled and the preadipocytes mature into new, viable tissue. The control tissue after four weeks exhibits a small region of healthy tissue near the boundaries of the tissue and a larger necrotic region as shown by the perilipin negative center. The center tissue morphology also suggests the formation of fibrotic tissue similar to observations by Choi et al detailing the increase in collagen fibers after grafting through 16 weeks (supplemental Figure 1) [28]. Similar to Kato et al., there is a low presence of ASCs observed in the center of the tissue [27]. In contrast, the pretreated grafts showed a larger amount of surviving adipocytes and remodeling from ASCs. The viability of the adipocytes is observed several hundreds of micron deeper into the tissue than that of the controls, and the remodeling is still ongoing as evident by the uneven morphology. The morphology of the pretreated grafts is more similar to the baseline tissue than the control fat graft, suggesting that the remodeling phase is progressing more rapidly. The decrease in fibrotic tissue in the pretreated grafts in comparison with the control grafts suggests there is less damaged adipose tissue four weeks after the initial graft.

### The Remodeling Phase is More Robust Following Helium-based Radiofrequency Pretreatment

**Fig. 2.**
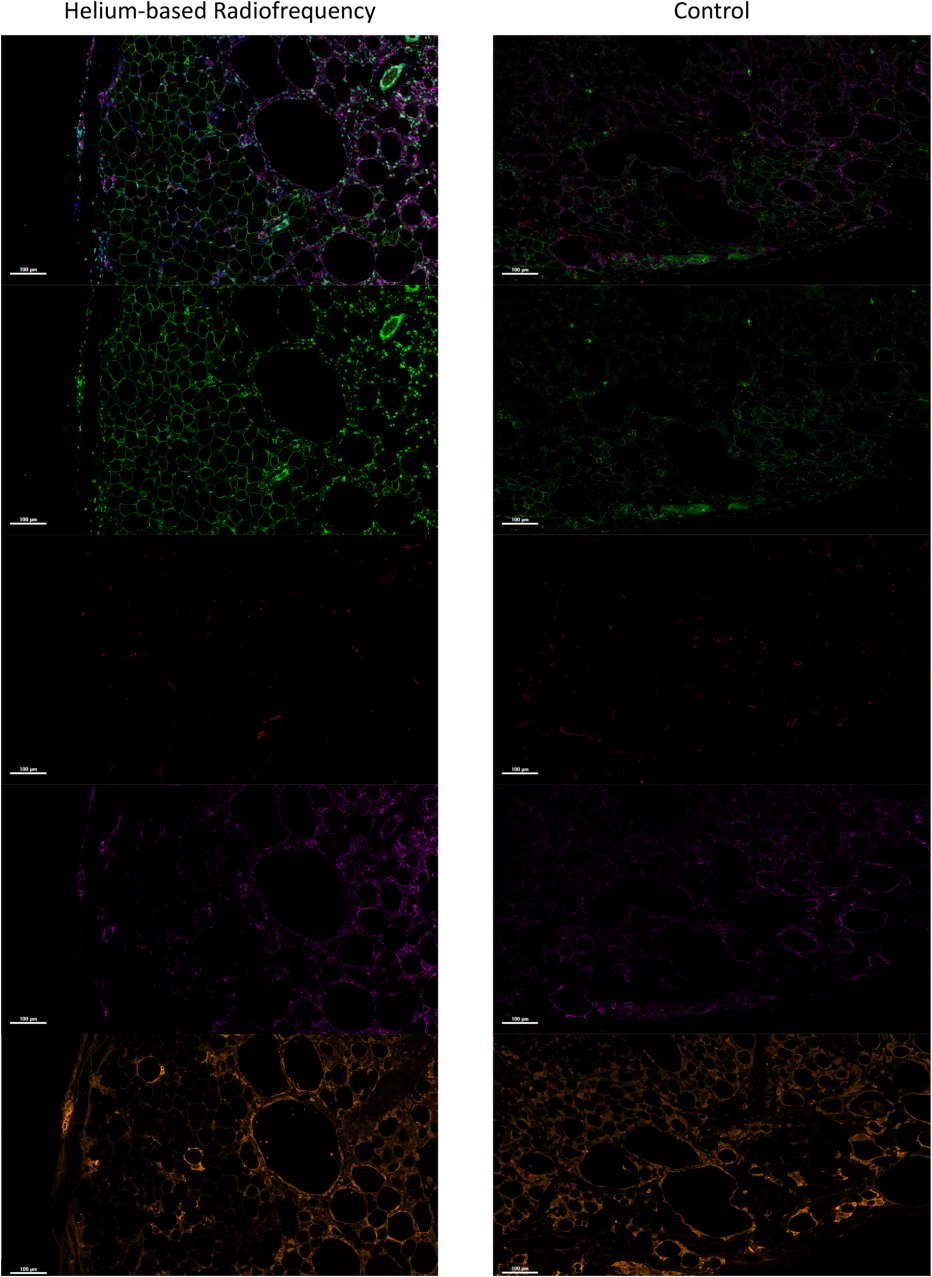
Helium-based radiofrequency pretreatment of the recipient tissue enhanced the adipose tissue remodeling 4 weeks after the initial graft. The merged image shows staining for fat viability (perilipin, green), vascularity (CD31, red), and an adipose derived stem cell surface marker (CD44, purple). The individual images for perilipin, CD31, and CD44 are shown below the merged image in row 2, row 3 and row 4 respectively. Macrophages are shown in the bottom row (MAC2, orange) from a different stained section of the same tissue. The fat graft with pretreatment (left column) exhibits stronger staining for viability and ASCs in comparison to the controls (right column). There is no difference in vascularity staining between Renuvion pretreatment and the control. The macrophages in the pretreated group are more localized in the remodeling tissue in contrast to their more uniform distribution in the control group.

The remodeling phase of fat graft retention requires the removal of dead adipocytes by macrophages, the proliferation of ASCs and their maturation into mature adipocytes, and the growth of new vascularity into the tissue. The results of these processes after four weeks are visualized in Figure 2. The top row shows the merged comparison between pretreated and control where the adipose viability (green, perilipin, second row), vascularity (red, CD31, third row), and ASCs (purple, CD44, fourth row) are compared in areas undergoing remodeling. The bottom row shows the presence of macrophages (orange, MAC2) stained on a separate section of the same tissue.

The remodeling of the adipose tissue was stimulated by the helium-based rf pretreatment in comparison with the controls. The adipose tissue in the pretreated graft retains the adipose morphology and stains perilipin positive for a few hundred microns into the fat graft, which indicates viable adipose tissue. Beyond these hundred microns, there are large lipid droplets without the surrounding adipocyte tissue, suggesting the accumulation of lipid droplets from dead adipocytes. The main difference between the pretreated and control grafts is the presence of strong perilipin-stained cells surrounding the lipid droplets. These cells colocalize with the staining for CD44, indicating that they are ASCs [29]. CD44 is a transmembrane glycoprotein and cell surface adhesion receptor that is present in many leukocytes and stem cells. CD44 signaling together with the presence of the strong perilipin staining indicates that these cells are viable ASCs [27, 30].

These viable ASCs exhibit stronger staining deeper into the tissue in the pretreated grafts. In combination with the viable mature adipocytes near the edge of the tissue, this observation suggests that the regeneration of the adipose tissue is ongoing deeper into the tissue. In addition, the macrophage staining intensifies deeper into the tissue, showing the active remodeling of tissue in comparison with the healthy adipose tissue in the first few hundred microns from the tissue edge. These macrophages have two functions that rely on their phenotype. The inflammatory M1 macrophages will remove the dead adipocytes and lipid droplets to allow for the ASCs to mature into healthy adipose tissue, while M2 macrophages will develop a cyst wall around the lipid droplets and fail to remodel the tissue [5]. The latter is finalized after the first three months of remodeling; thus, it is too early to diagnose which macrophage function is occurring after four weeks.

The control fat graft does not exhibit similar characteristics to the pretreated fat graft. Viable staining and adipose morphology quickly disappear about 100 microns away from the tissue edge. There is a small fraction of cells that are viable ASCs as depicted by the strong perilipin staining in combination with the CD44 stain; however, it is marginal and indicates that the remodeling phase is not recruiting as many ASCs into the tissue as the pretreated tissue. Furthermore, macrophage staining is present throughout the tissue instead of more localized, which suggests most of the dead adipocytes remain and there is not a large viable adipose tissue zone. The vascularity of the tissue as visualized by CD31 (Fig. 2) and VEGF (Supplemental Figure 2) shows similar staining to the pretreated fat grafts; thus, there is no observed difference in vascularity or signaling for angiogenesis between the samples.

**Fig. 3.**
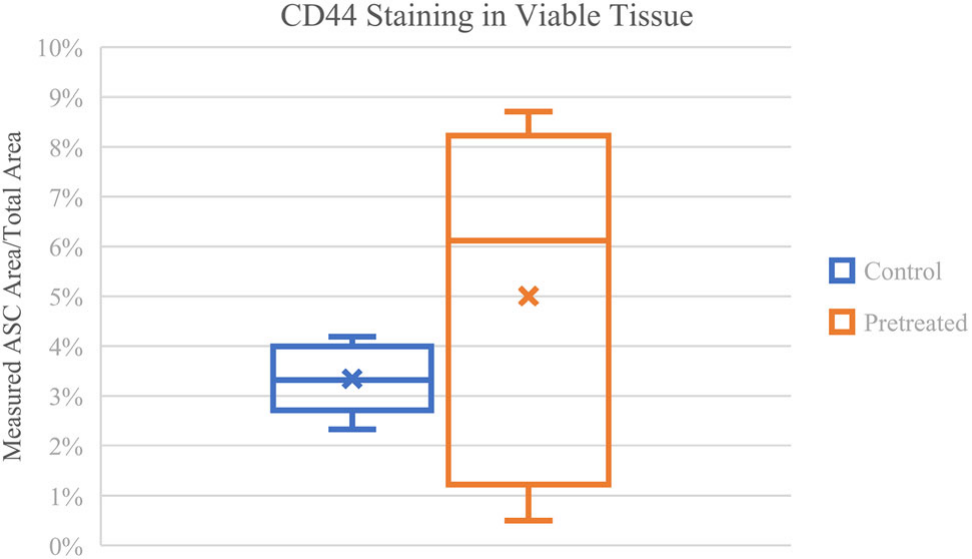
Recipient pretreatment (n=5) increased the presence of ASCs into a bolus fat graft after four weeks in comparison to the control (n=5). The area of viable adipose tissue was measured in QuPath after spectral unmixing with Phenochart. The ASC marker was measured using a pixel classifier to measure ASC signal in areas with viable tissue. On average (middle line), pretreatment increased the signal of the ASC marker, indicating increased presence of ASCs.

The increase in viable tissue CD44 staining in the pretreated arm compared to the control is shown in Fig. 3. The images were spectrally unmixed, the viable regions were annotated, and a pixel classifier was applied in QuPath for CD44 staining. On average, the pretreated group (5.0%) increased the staining when compared to the control group (3.3%). The variance in the pretreated data was high, with three samples between 6-8% and two samples between 0.5 and 2%. These results suggest a higher concentration of ASCs in the pretreated fat grafts in comparison with the control.

## Discussion

The use of an energy-based device for stimulation of the recipient tissue was investigated for increased retention of fat grafts. Although new fat grafting techniques and animal models show increased retention using smaller fat grafts over a bolus fat graft model, the latter was used to assess if helium-based rf pretreatment could affect the remodeling of fat with large necrotic tissues in the center [31, 32]. This model allows for the assessment of the depth of effect, which in this study is shown to be improvement of at least a few hundred microns into the tissue at 4 weeks. The cranial fat graft model was selected and modified from Kato et al. because the fat was grafted in a site with minimal native fat and was easily accessible [27]. While typical animal retention studies assess fat grafts between 12 weeks and one year, the mechanisms of adipose tissue regeneration and the effects from energy-based pretreatment occur on shorter times scales. Four weeks were chosen because Kato et al. observed the highest concentration of ASCs at this time point [27]. The results discussed here will need to be proven in larger animal models and clinical studies to project any applicability of recipient site pretreatment using a helium-based rf pretreatment for large volume fat grafting. This results of this study and future animal studies will have limited direct applicability to humans; however, the results will build a foundation for potential mechanisms of action to pursue in clinical trials.

The results suggest that helium-based rf pretreatment leads to an increased viability of the fat grafts through higher concentrations of viable ASCs present in the tissue. These smaller, strongly perilipin positive cells localized near macrophages indicate that the ASCs are migrating to the locations where dead adipocytes and lipids are removed. The apparent increase in viable ASCs may be due to enhanced proliferation or paracrine recruitment of these cells in response to tissue pretreatment.

As shown by several groups, helium plasma treatment of ASCs in vitro resulted in increased proliferation of ASCs [23, 33]. Furthermore, helium plasma treatment stimulated ASCs by upregulating genes for stem cell proliferation while downregulating intrinsic apoptotic pathways and by activating ERK1/2, Akt and NF-kB through nitric oxide production [23, 34]. These activated signaling pathways in response to helium plasma are similar to pathways in ASCs stimulated by PRP [11]. The helium plasma produced by the helium-based rf device has similarities to other helium plasma devices described as cold atmospheric plasma (CAP), namely the production of reactive oxygen and nitrogen species (RONS), ultraviolet light, and electric field gradients [35]. The plasma discharge is created when rf energy delivered to the electrode is transferred to the helium gas, which creates an electrically conductive plasma. As this discharge interacts with medical solutions, such as saline or Ringer’s, the charged molecules in the plasma dissociate the liquid molecules and some recombine into bioactive RONS. Therefore, the enhanced recruitment or proliferation of ASCs in the grafted adipose tissue may be due to the RONS produced by the helium-based rf plasma and may result in similar stimulatory properties to PRP. Future studies are needed to tie these potential mechanisms of action to pretreatment of the recipient site and increased presence of ASCs in pretreated grafts.

The response in the recipient tissue to pretreatment may exhibit enhanced immune cell function as observed by cold plasma treatment [36, 37]; however, the pretreatment does not show any evidence for influencing angiogenesis into the tissue. In comparison with the study by Kato et al., the remodeling zone of the control groups looks similar at 4 weeks, and however, the helium-based rf pretreated groups resemble remodeling at 8 weeks [27]. Furthermore, there was less fibrotic tissue observed in the pretreated grafts in comparison with the control grafts. These observations suggest that either the biological effects of this pretreatment result in a hastened remodeling of the tissue, or that there are more immune cells recruited to the tissue that may contribute to the observed healthier tissue. However, immune cell function was not measured, and more studies are needed to prove recruitment of immune cells or enhancement of function contributed to the healthier adipose tissue in pretreated grafts. CD44 has a role in both pro- and anti-inflammatory environments for regulating immune cell migration and recruitment [30, 38, 39] and its higher signal in the pretreated fat grafts may influence the rate of adipose tissue remodeling. Unlike other reports suggesting increased angiogenesis due to rf energy or CAP treatment [22, 40, 41], the helium-based rf pretreatment of the tissue did not stimulate angiogenesis into the fat graft. This may be due to the difference in heat delivery to the tissue (rf energy), RONS generation and delivery to the tissue (plasma), or the differences in CAP devices. This also suggests that treatment using a helium-based rf device may stimulate the tissue differently than other energy-based devices, specifically low-level laser therapy (CO_2_ fractional lasers) where increased angiogenesis was observed post-treatment in vitro [7].

The mechanism of action behind the increased viability of the fat grafts with pretreated recipient sites will need more detailed investigations to show the role of recipient cells and donor graft cells. An extension of this model using native and green fluorescent protein expressing mice was utilized by Doi et al. to show that mature adipocytes mostly derived from the donor tissue ASCs after 12 weeks of remodeling [42]. For vascularity, the mural cells and nearly half of the vascular endothelial cells originated from the donor and recipient bone marrow, respectively. The recipient immune cells were shown to infiltrate into the areas of necrotic adipose tissue. These results serve as a suitable model to further investigate if the helium-based rf pretreatment affects the immune cell recruitment and ASC replacement into the tissue. The time points to investigate would also need to change from the current study (4 weeks) to measure promotion of angiogenesis and recruitment of immune cells (1-2 weeks) the long-term fat retention and formation of necrotic cysts (12 weeks).

## Conclusion

This work is the first step in understanding the impact of helium-based radiofrequency pretreatment on tissue in anticipation of grafting fat, confirming what has been observed clinically. The stimulated tissue and cellular responses are consistent with other enhanced graft survival optimization techniques. The pretreatment increased the viability of the adipose tissue four weeks after the fat graft procedure. The results suggest the increase in viability is due to the higher concentration of ASCs in the tissue. The mechanism may be through either proliferation or recruitment by paracrine signaling. Future work needs to consider the complete impact of helium-based radiofrequency pretreatment for fat graft retention by investigating final retention volume after remodeling is completed, the mechanisms behind proliferation and migration of ASCs, and how this treatment would interact with native adipose tissue in the recipient site.

### Supplementary Information

Below is the link to the electronic supplementary material.

Supplementary file1 (PNG 3342 kb)

Supplementary file2 (PNG 7389 kb)
