# Correction: Enhanced Fat Graft Viability and Remodeling Using a Helium-based Radiofrequency Device to Prepare the Recipient Site

**DOI:** 10.1007/s00266-024-03853-1

**Published:** 2024-01-31

**Authors:** Paul G. Ruff, Aris Sterodimas

**Affiliations:** 1 West End Plastic Surgery, MedStar Georgetown University, Washington, DC USA; 2 Department of Plastic and Reconstructive Surgery, Metropolitan General Hospital, Athens, Greece

**Correction to: Aesth Plast Surg** 10.1007/s00266-023-03749-6

The original online version of this article was revised to correct the presentation of the name of first author Paul G. Ruff IV.

The original article has been corrected.

