# Supplementary figures and images for: Enhanced Fat Graft Viability and Remodeling Using a Helium-based Radiofrequency Device to Prepare the Recipient Site
Source: Aesthetic Plast Surg. 2023 Dec 14;48(4):612–20. doi: 10.1007/s00266-023-03749-6 (PMC10954941; doi:10.1007/s00266-023-03749-6)

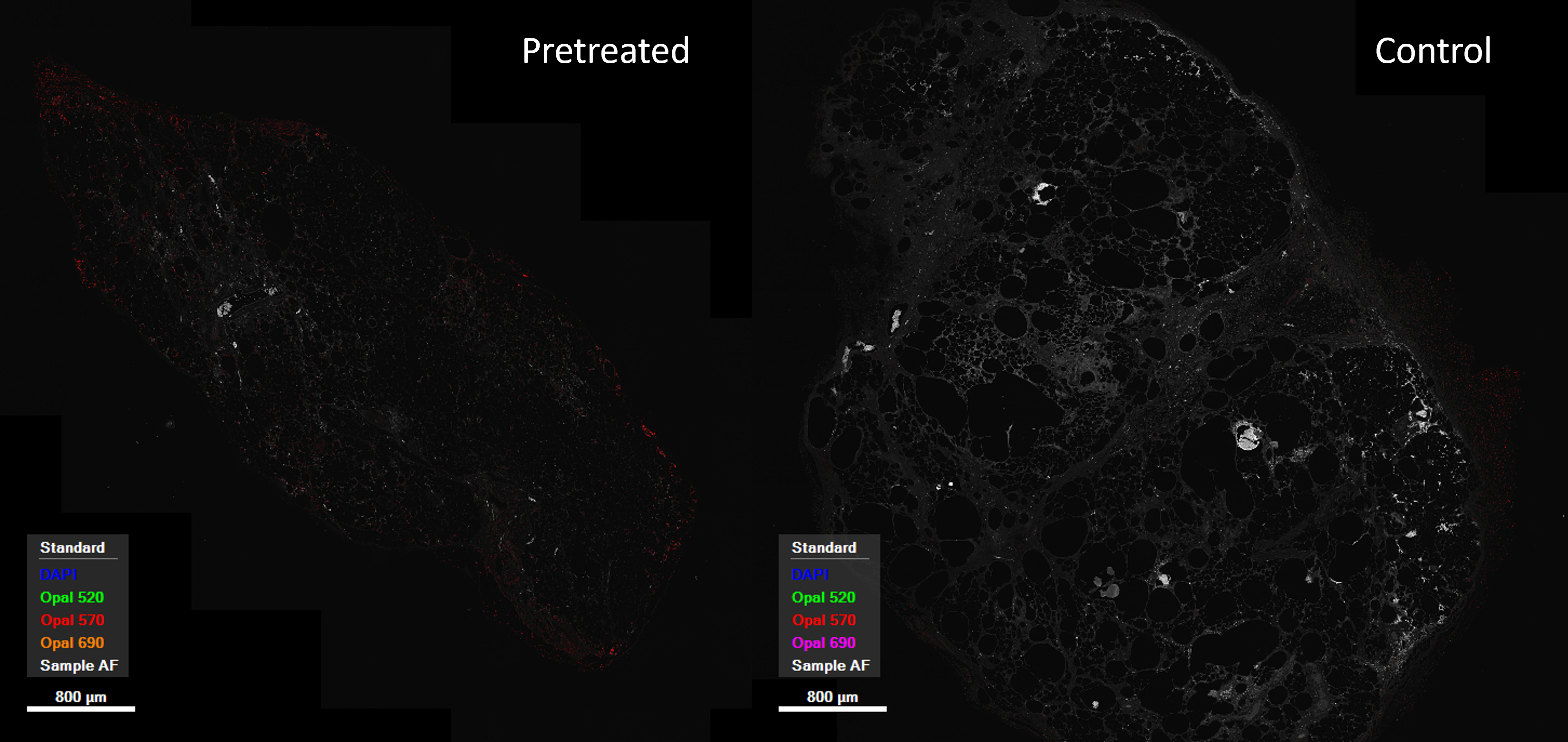

Supplement: Supplementary file 1 — Supplementary file1 (PNG 3342 kb) [file 266_2023_3749_MOESM1_ESM.png]

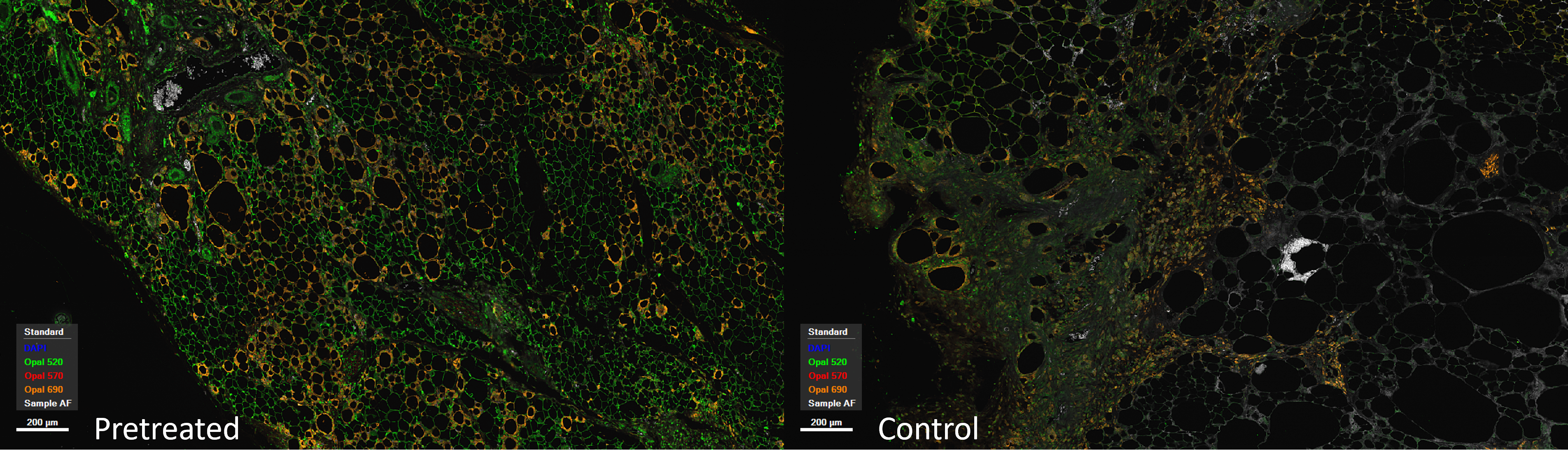

Supplement: Supplementary file 2 — Supplementary file2 (PNG 7389 kb) [file 266_2023_3749_MOESM2_ESM.png]
